# Post-Cardiac arrest targeted temperature management in a parturient with severe COVID-19 disease

**DOI:** 10.12669/pjms.39.4.7193

**Published:** 2023

**Authors:** Ali Eman, Onur Balaban, Kezban Özmen Süner, Bora Özgün

**Affiliations:** 1 Ali Eman Department of Anesthesiology and Reanimation, Sakarya Training and Research Hospital, Sakarya Turkey; 2 Onur Balaban Associate Professor, Department of Anesthesiology and Reanimation, Sakarya University, Sakarya, Turkey; 3 Kezban Özmen Süner Department of Intensive Care, Sakarya Training and Research Hospital, Sakarya Turkey; 4 Bora Özgün Vitale Obstetrics and Gynecology, Hospital Antalya, Turkey

**Keywords:** Targeted Temperature Management, Therapeutic Hypothermia, Pregnant, Cesarean Section, Covid-19, Cardiac Arrest, Postpartum

## Abstract

**Background and Objective:**

Targeted temperature management (TTM) may improve neurological outcomes and mortality after cardiac arrest. We present a targeted mild hypothermia treatment in a postpartum patient with COVID-19 after successful cardiopulmonary resuscitation (CPR).

**Case presentation:**

A 23 year old, 26-week pregnant patient with the diagnosis of COVID-19. The patient developed respiratory arrest followed by cardiac arrest and underwent CPR for six minutes. The patient underwent an emergency cesarean section after CPR in intensive care unit. After the resuscitation, 72-hours hypothermia protocol was initiated. We extubated the patient 13 days after the hypothermia procedure. The patient was conscious and cooperative. Respiratory distress worsened in the following days; the patient was re-intubated 18 days after the TTM. The benefit of targeted hypothermia was improved neurologic outcome in our patient. However, severe infectious complications led to multi-organ failure and the patient died on the 45th ICU admission day.

## INTRODUCTION

Clinical usage of targeted temperature management (TTM) may decrease metabolic demand for end organ preservation in critical ill patients. Targeted-hypothermia treatment may preserve brain functions and improve neurological outcomes in patients whose spontaneous circulation returns after cardiac arrest.^1^ Although there is an assumption of reduction of cerebral oxygen consumption, the exact mechanism for the effect of this procedure is not clear.^2^

TTM may also improve the hypermetabolic state in COVID-19 critical illness by reducing the metabolic rate and may improve hypercapnia and hypoxia which was demonstrated in a case series recently.^3^ There is anecdotal use of this method in a pregnancy; however, there is no report in the literature about the postpartum implementation in pregnancy with COVID-19.^4^,^5^ We report targeted-mild-hypothermia treatment after postpartum cardiac arrest in a pregnant woman who was followed up in our intensive care unit with severe COVID-19 infection.

## CASE PRESENTATION

Informed consent was obtained from the relatives of the patient. Twenty three years old, 26-weeks pregnant was admitted to the obstetrics emergency clinic with complaints of cough, vomiting and diarrhea. The medical history of the patient included Type-1 diabetes. The patient had a (+) PCR test for COVID-19, and due to the complaints of tachypnea, dyspnea and low oxygen saturation she was transferred to the intensive care unit on the 4th day of her admission to the obstetrics department. Her peripheral oxygen saturation was between 92% and 97% under oxygen therapy using a reservoir mask at 15 L/min flow. We administered non-invasive ventilation with intermittent high flow nasal oxygen (HFNC) and continuous positive airway pressure (CPAP).

On the 2nd ICU day, respiratory and cardiac arrest occurred. We endotracheally intubated the patient, and we performed cardiopulmonary resuscitation (CPR) for about six minutes. A bedside emergency cesarean section operation was performed in the intensive care unit. The baby was born with an APGAR score of three and four at the 1st and 5th minutes, respectively, and the mother’s surgical procedure was completed without complications. The patients Glascow coma score (GCS) was 3/15 after the CPR, before the initiation of TTM. Approximately six hours after resuscitation, target-controlled hypothermia treatment (Arctic Sun^®^ Medivance Corp, Louisville, Co.) was carried out in three stages. The main indications of TTM were the it was a witnessed arrest and the increase of oxygen demand and decrease of the oxygen delivery in the patient.

In the first stage, cooling was applied with a target body temperature of 34.5-35°C within six hours. In the next step, the body temperature was kept in the range of 34.5-35°C for 24 hours. In the last stage, the body temperature was increased by 0.2–0.3°C per hour until the final body temperature was constant at 37 to 37.5°C. Midazolam, remifentanil, and rocuronium infusions were given to the patient during the hypothermia process.

On the 7th day, the patient developed a sudden decrease of oxygen saturation, and then cardiac arrest occurred, and we performed three minutes CPR. On the 14th day of ICU admission, transbronchial aspirate sample showed a negative COVID-19 PCR test. There was an improvement in oxygenation (PaO_2_/FiO_2_ ratio increased to 180 from 130). There was also improvement in CO_2_ removal as the arterial PCO_2_ was 56.7 mmHg before TTM and was 62.1 mmHg on the first day of TTM and decreased to 53.9 mmHg on the following day and was 46.8 mmHg on the 4th day from the initiation of TTM. We extubated the patient on the 15th day in ICU. She was conscious, cooperative, and well orientated (GCS: 15/15). We continued oxygen support using a face mask with a reservoir. We did not experience any major hemorrhagic complications after the operation. We monitored bleeding with frequent hemogram and blood drainage follow-ups. The patient’s coagulation, hemogram and other laboratory parameters before and after hypothermia application are given in Table-I.

**Table-1:** The laboratory values before and after the hypothermia procedure.

Laboratory test (normal range reference)	Admission to intensive care	Before hypothermia	24th hour of hypothermia	48th hour of hypothermia	End of hypothermia	5th day after hypothermia	15th day after hypothermia	30 days after hypothermia	Value on the day the patient died
AST (0-35 U/L)	36	53	34	39	26	45	22	11	19
ALT (0-35 U/L)	14	18	13	17	16	32	22	7	11
LDH (0-247 U/L)	410	908	600	756	673	785	460	291	309
CRP (0-5 mg/L)	62	119	161	173	115	48	56	130	260
Procalcitonin (< 0.5 ng/ml)	0.06	1.28	1.70	1.14	0.64	0.82	0.09	1.32	4.10
Ferritin (5-204 mcg/L)	48	62	63	78	94	264	202	986	5552
D-dimer (0-500 ugFEU/L)	799	2600	4060	3320	1790	2540	1580	3180	4620
EGFR (>90 mL/dk)	132	122	113	70	78	125	143	65	100
Creatinine (0.51-0.95 mg/dl)	0.5	0.68	0.74	1.1	1	0.63	0.46	0.72	0.82
Leukocyte (4.6-10.2 K/uL)	20.2	33.2	18	24.8	25.8	30.7	18.1	16.9	15.9
Hematocrit (37.7-53.7 %)	28.6	26.2	27.2	27.3	24.8	26.4	30	24.2	27.5
Thrombocyte (142-424 K/uL)	300	317	142	193	263	462	427	454	175
Lymphocyte (0.60-3.4 K/uL)	1.78	1.41	0.58	1.11	0.61	2.02	2.2	1.80	1.44
PT (7-13 s)	8.9	10	9.6	10.4	10.4	13.2	11.9	11	12.1
aPTT (18-33 s)	26.6	31.6	3.9	36.8	30.5	27.6	26.7	29.9	31.3
INR (0.8-1.3)	0.85	0.95	0.90	0.99	0.99	1.26	1.14	1.05	1.15

**AST:** aspartate aminotransferase, **ALT:** alanine aminotransferase, **LDH:** lactate dehydrogenase, **CRP:** C reactive protein, **PT:** prothrombin time, **aPTT:** active partial thromboplastin time, **INR:** international normalized ratio, s: second

On the 17th day, respiratory distress occurred again, and blood CO_2_ levels increased. The patient was re-intubated on the 19th day, and a percutaneous tracheostomy was performed on the 23rd day in ICU. The patient developed cardiac arrest on the 28th and 37th days, and she died on the 45th day of her admission to the ICU due to severe sepsis and multi-organ failure.

## DISCUSSION

In this case, targeted-mild hypothermia treatment preserved the neurologic functions after cardiac arrest in a postpartum patient with severe COVID-19. The results regarding the positive effect of TTM application on neurological recovery after cardiac arrest are controversial. Systemic hypothermia improves neurological outcomes and mortality in patients whose spontaneous circulation returns after cardiac arrest; however, there are studies showing that it has no effect.^2^,^6^ The European Resuscitation Council and the European Intensive Care Societies jointly published a guideline in 2021 and recommended target-controlled hypothermia in cases of in-hospital and out-of-hospital cardiac arrest, regardless of the initial rhythm.^7^

A draft consensus statement by the society for Obstetric Anesthesia and Perinatology in 2014 also recommended TTM, which should be strongly considered after cardiac arrest in obstetric or postpartum patients whose indications are similar to general population.^8^ Studies reporting that TTM treatments do not provide significant neurological improvement mostly include patients having out-of-hospital cardiac arrest.^6^ For this reason, in-hospital witnessed arrests and relatively short resuscitation times are important aspects for the success of the TTM.

In one case report, including two cases of postpartum TTM, one of the patients was discharged without complications, while the other patient was transferred to intensive care in a vegetative state.^9^ Patient one was 31 years old and 39 weeks pregnant. Cardiac arrest occurred two minutes after delivery by cesarean section, and spontaneous circulation started after approximately four minutes of CPR. TTM was started with a target temperature of 33°C ± 1°C for 24 hours. The patient was extubated on the 4th day of her admission to the intensive care unit, and was discharged on the 10th day without any complications. Our patient had CPR for six minutes before the hypothermia procedure, which may be considered a relatively short time, similar to this patient.

The second patient was 30 years old whose spontaneous circulation started after 40 minutes of CPR. TTM was applied to the patient at 33°C, and the patient was transferred to the care center without neurological improvement on the 21st day of hospitalization. In this case, pulmonary thromboembolism accompanied to cardiac arrest. Serious comorbid conditions accompanying cardiac arrest may affect the success of neurologic recovery in TTM. Prolonged CPR times, improperly performed CPR, and the primary cause of cardiac arrest may also affect neurological outcomes.

In previous studies, target temperature values were mainly between 32 and 36°C.^10^ It has been reported that if the target temperature is kept around 36°C, there is a decrease in the undesirable adverse effects of hypothermia; at the same time, there is a statistically insignificant difference in neurological recovery.^10^ In patients with COVID-19, moderate therapeutic hypothermia may be beneficial in reducing the oxygen requirement with its anti-inflammatory effect and reduce metabolic needs and may improve refractory hypercarbia and hypoxic conditions.^3^ Thus, we preferred a mild-hypothermia for TTM in our patient with a target temperature of 34.5-35°C.

### Authors Contribution:

**AE**: conceived, designed, writing and editing of manuscript, is responsible for integrity of research.

**KOS and BO:** did data collection and manuscript writing

**OB:** did review and final approval of manuscript.
